# Development of a healthy biscuit: an alternative approach to biscuit manufacture

**DOI:** 10.1186/1475-2891-5-7

**Published:** 2006-03-15

**Authors:** WJ Boobier, JS Baker, B Davies

**Affiliations:** 1 Health and Exercise Science Research Unit, School of Applied Sciences, University of Glamorgan, Pontypridd, CF37 1DL, UK

## Abstract

**Objective:**

Obesity (BMI >30) and related health problems, including coronary heart disease (CHD), is without question a public health concern. The purpose of this study was to modify a traditional biscuit by the addition of vitamin B_6_, vitamin B_12_, Folic Acid, Vitamin C and Prebiotic fibre, while reducing salt and sugar.

**Design:**

Development and commercial manufacture of the functional biscuit was carried out in collaboration with a well known and respected biscuit manufacturer of International reputation. The raw materials traditionally referred to as essential in biscuit manufacture, i.e. sugar and fat, were targeted for removal or reduction. In addition, salt was completely removed from the recipe.

**Participants:**

University students of both sexes (n = 25) agreed to act as subjects for the study. Ethical approval for the study was granted by the University ethics committee. The test was conducted as a single blind crossover design, and the modified and traditional biscuits were presented to the subjects under the same experimental conditions in a random fashion.

**Results:**

No difference was observed between the original and the modified product for taste and consistency (*P > 0.05*). The modified biscuit was acceptable to the consumer in terms of eating quality, flavour and colour. Commercial acceptability was therefore established.

**Conclusion:**

This study has confirmed that traditional high-fat and high-sugar biscuits which are not associated with healthy diets by most consumers can be modified to produce a healthy alternative that can be manufactured under strict commercial conditions.

## Background

The UK diet is high in fat, particularly saturated fat, e.g. in 1992 over 40% of all energy intake was derived from fat, with over 15% coming from saturated fat alone [1]. The European Economic Community (EEC) nutrition policy goal suggested that only 20–30% of energy should be derived from fat [2]. There is considerable epidemiological evidence that nutritional intake is related to the risk of coronary heart disease (CHD) [3,4]. To reduce the incidence of CHD the department of Health has recommended that the proportions of energy derived from total fat should be reduced to 35%, and from saturated fat to 10% [1].

Also, the replacement of saturated or trans-unsaturated fat with unhydrogenated unsaturated fats is associated with larger reductions in risk factors associated with CHD than replacement by carbohydrate [5].

Other dietary changes considered helpful in the fight against coronary heart disease include consumption of a high fibre diet [6], reduction of salt and sugar intake [7-9]. In a study conducted by Pietinen *et al*., [6] the mean intake of soluble fibre in the diet was 5.4 g.day^-1 ^compared to 18.9 g.day^-1 ^for insoluble fibre. They reported that an increase in daily soluble fibre by 3 g reduced the risk of coronary death by 27%. Dietary sodium reduction has been recommended to reduce hypertension and cardiovascular disease mortality and morbidity [9]. Large amounts of sucrose, or other refined carbohydrate, cause an increase in lipid triglyceride, an increase not associated with complex-starch intake [8]. Biscuits are a popular food eaten by both children and adults; however, they are typically high in the materials (fat and sugar) that make them "unhealthy". In the manufacture of biscuit dough, it is traditional to use fat which is semi-solid at room temperature e.g. palm oil which contains 50% saturated fatty acids. In addition, the biscuit market is dominated by short dough biscuits having fat levels in excess of 20% [10]. Biscuits are therefore an obvious choice when consumers are asked to reduce their total fat intake. The concluding paragraph of the Diet and Heart Disease report [11] states that high fat bakery products should be reduced in the diet. In addition, biscuits have previously been highlighted in a report by Willet *et al*., [12] as being significantly associated with an increased risk of CHD.

The development of a commercially viable biscuit attractive to children and adults that will have a significant reduction in fat and sugar, with fewer calories and contain nutrients designed to reduce the risk of coronary heart disease is highly desirable.

The purpose of this study is to describe the development of a healthy biscuit (functional food) with low moisture, long shelf life that has commercial viability, has had sodium chloride reduced and pre-biotic fibre, B6, B_12_, folic acid and vitamin C added.

### Functional food

A food can be regarded as functional if it is satisfactorily demonstrated to beneficially affect one or more target functions in the body, beyond adequate nutrition, in a way that improves health and well being or reduces the risk of Disease [13,14]. The term functional food was first used in Japan [15], and Japan has been active in the development of this type of product since the early 1980's.

Research to date has focused on several factors that contribute to the health status of individuals. This study has focused attention on the modification of a standard biscuit by the addition of vitamin B_6_, vitamin B_12_, Folic Acid, Vitamin C and Prebiotic fibre, while reducing salt and sugar, thereby converting a traditional food product into a functional one.

### Vitamin C (ascorbic acid)

Ascorbic acid (vitamin C, 2,3-didehydro-L-threo-hexonic acid-g-lactone) is an acid derivative of a 6-carbon sugar. The presence of the "diol" grouping at carbons 2- and 3- enables the vitamin to act as a powerful reducing agent. It is evident that subjects with acute myocardial infarction have lower plasma vitamin C than control Subjects [16-19]. Although there is no universally accepted recommended daily intake of vitamin C, the World Health Organisation recommends a daily intake of 30 mg, whereas in the UK the RDA is 60 mg^1^.

### Prebiotic fibre (fructo-oligosaccharide)

A prebiotic is defined as a non digestible food ingredient that beneficially affects the host by selectively stimulating the growth and/or the activity of one or a number of bacteria in the colon that have the potential to improve health [20,21]. Prebiotics, most often referred to as non-digestible oligosaccharides, are extracted from natural sources, e.g. Inulin and oligofructose, or synthesised from disaccharides. Actilight^®^, the prebiotic that was used in this study was obtained from sugar beet comprising of one molecule of glucose linked to two, three or four molecules of fructose. Its use in the final product formulation contributed to the dough characteristics and the organoleptic properties of the biscuit.

### Vitamin B_6_

A number of studies have shown biochemical signs of deficiency or inadequacy of vitamin B_6 _in around 10–20% of apparently healthy people [22,23]. This may be a factor in the development of atherosclerosis, a result of impaired metabolism of methionine and raised circulating levels of homocysteine [22,23]. Reference nutrient intakes (RNIs) are based on 15–16 μg.g^-1 ^of protein per day. At average intakes of 100 g protein per day this gives an RNI of 1.5–1.6 mg of vitamin B_6. _Average intakes in Britain are between 20–30 μg.g^-1 ^protein ^23^.

### Vitamin B_12_

The current RNI for vitamin B_12 _is 1.5 μg^.^day^-1 ^for males and females 15 to 50+ years [1]. The addition of Vitamin B_12 _in the modified biscuit follows the recommendations of several authors [22-24], who observed elevated homocysteine concentrations as a result of impaired vitamin B_12 _status which has been implicated as a cause of premature cardiovascular disease.

### Folate (folic acid)

Folic acid is involved in a number of single carbon transfer reactions, e.g. in the synthesis of methionine [25], the metabolism of which results in the production of homocysteine as an intermediate product. Normal levels of homocysteine for men are around 8–12 micromoles per litre (μmol.l^-1^) and for women normal levels are 6–10 μmol.l^-1 ^[26]. High blood homocysteine is thought to be an independent risk factor for CHD [26-29]. The conventional view is that as an individual regresses into a negative folate balance, there is a reduction in plasma and tissue concentrations. This would be followed by small and eventually large increases in homocysteine levels [30].

Since moderate reduction in folate status results in elevated plasma homocysteine, a relationship between folate status and risk of cardiovascular disease has been established [29,31]. Whilst supplements are suitable for those aware of the potential benefit, even following significant publicity, it's clear that only a small minority of people, especially in the poorer socio-economic sector where the risks may be greater, will take beneficial supplements on a regular basis [32]. This has resulted in suggestions that the diet should be fortified with folic acid, and in the USA it is now policy that flour is required by law to be fortified with folic acid.

## Methods

Development and commercial manufacture of the functional biscuit was carried out in collaboration with a well known and respected biscuit manufacturer of International reputation. The raw materials traditionally referred to as essential in biscuit manufacture, i.e. sugar and fat, were targeted for removal or reduction. In addition, salt was completely removed from the recipe. Fat and sugar was substituted for other materials not associated with poor health, and are outlined later. Following initial manufacturing difficulties, which included poor release from the rotary moulder and tailing, sugar and salt was successfully removed from the recipe of the biscuit base and fat was significantly reduced. The final product was produced under strict commercial conditions to confirm manufacturing viability, and then assessed organoleptically to confirm consumer acceptance.

### Assessment of the modified product

As far as the consumer is concerned, the most important part of any product is taste perception and eating qualities. University students of both sexes (n = 25, with a mean age ± 1 standard deviation (SD) of 23 ± 2 yrs) having read, completed and signed informed consent forms agreed to act as subjects for the study. Ethical approval was granted by the University ethics committee.

The test was conducted as a single blind crossover design, over a four week period, over two testing periods of two weeks duration. The modified and traditional biscuits were presented to the subjects under the same experimental laboratory conditions in a random fashion with one week intervening between trials. Subjects were given two biscuits per day including weekends. Prior to biscuit consumption, all subjects confirmed that they had not eaten for two hours and were asked to swill out their mouths with cool fresh water to enhance taste perception. All testing for all subjects was performed between the hours of 11 and 12 am. The experimental environment was kept constant for both conditions and no outside influences were allowed to interfere with the subject's assessments of the products (smells, music, temperature etc). The trial was repeated one week later for a further two weeks following the same experimental conditions. A hedonic scale 0–9 was used to score the biscuits taste, a score of 1 representing "dislike very much" and 9 representing "like very much". The participants were asked to comment briefly on the reasons for the responses given for either the modified or standard product.

Statistical analysis and commercial acceptability

Statistical analysis was performed using computer software (SPSS). A Wilcoxon Matched Pairs Signed Rank Non-Parametric Test was used to establish the degree of significance between products. Non parametric statistics were used was because of lack of normal data distribution observed following repeated Kolmogorov-Smirnov tests. Significance levels were accepted at *p *< 0.05.

## Results

No difference was observed between the original and the modified product in both trials using the hedonic scale (*p *> 0.05). The values recorded were 7.6 ± 1 for the traditional product and 7.4 ± 0.6 for the modified biscuit. The modified biscuit was acceptable to the consumer in terms of eating quality, flavour and colour. Commercial acceptability was therefore established. Product texture of the modified biscuit was a little more open than the standard biscuit, which was thought to be desirable in terms of eating quality. Moisture was maintained as target and there were no signs of ovality (distortion of the biscuit following baking). The product was sent through the manufacturing plant and processed at all stages without any problems. Samples were collected for analysis of vitamin content, other nutritional information and sensory evaluation.

### Commercial viability of the modified product

Target manufacturing bulk was achieved, and surface colour was consistent with the standard biscuit.

Biscuit bases were also checked against the standard product for hardness, using a Stevens Texture Analyser, and no differences were recorded. Results of vitamin analysis and nutritional information are detailed in Table 1.

**Table 1:** Nutritional Information for the Standard and Modified Product per 100 g of mixture. Zero indicates no concentrations of vitamins in the standard product

	Standard Product		Modified Product		
Energy	1869 kJ/448 kcal		1675 kJ/396 kcal		
Protein	4.8		6.0		
Carbohydrates	68.8		77.3		
Of which is sugars	32.5		22.5		
Fat	16.7		7		
- saturates	7.4		3.1		
- mono-unsaturates	6.8		2.42		
- poly-unsaturates	1.9		1.13		
Fibre	1.7		3.4		
Sodium	0.2		0.1		
	Per 100 g	Per biscuit	Per 100 g	Per biscuit	%RDA
Vitamin C	Zero	Zero	302 mg	55 mg	92
Vitamin B_6_	Zero	Zero	55 mg	2.0 mg	100
Folic Acid	Zero	Zero	1900 μg	347 μg	174
Vitamin B_12_	Zero	Zero	30.0 μg	2.7 μg	135

## Discussion

Biscuits are typically high in both fat and sugar and have been identified as a food contributing to negative health [1]. It has been recommended that the intake of biscuits and related products should be reduced [4,11]. This study concentrated on one of the best selling biscuits in the UK that is consumed regularly by both children and adults. An essential part of this study was the maintenance of commercial and consumer viability, i.e. the final product had to be produced using commercial parameters, and had to be acceptable to the consumer in terms of organoleptic properties. Reducing the materials that were thought to be essential in the standard product was difficult. The complex interaction between these raw materials necessitated a large number of formulation modifications, resulting in complicated manufacturing issues. For example, reduction in fat resulted in several undesirable effects such as toughening of base dough, shrinkage of final product, loss of colour and eating quality. Addressing one problem often created another.

### Biscuit dough

It was clear that dough's produced under laboratory conditions were significantly different when produced commercially. Specifically, dough water levels needed to be higher in the laboratory than on plant in order to facilitate processing by hand. Plant processed dough's needed to be drier to facilitate extraction from the rotary moulder. Benefits of reduced recipe water required for commercially processing biscuits include reduced hydration of flour proteins (less toughness of the dough) and less water to remove during baking in order to achieve the required moisture levels. It was clear that in order to maintain workable dough following fat reduction, that an increase in water was needed. Fat makes a significant contribution to dough consistency, with dough's becoming less short (tougher) with reduced recipe fat. This resulted in greater shrinkage during baking when the two biscuits were compared. The most important function of fat in biscuit dough is the prevention of gluten development which enhances eating quality [33]. Reduction in fat also resulted in the need to alter baking conditions in order to maintain a desirable product colour. This was achieved by increasing baking time and/or temperature. As a result of this, the eating characteristics of the product became very tough and could not be described as "melt-in-the-mouth". To reduce this toughening effect, dilution of flour proteins by the substitution of a portion of the flour with heat treated flour was made.

### Baking process and the modified product

During heat treatment, the flour proteins have been denatured and therefore unable to produce gluten. In this way, an original gluten forming protein content of 9% was reduced to 4.5%. In addition, the use of emulsifier extended the functionality of the available fat. A modification in the mixing parameters was also made to reduce the toughening during the mixing phase. The combination of these modifications contributed to the viability of the final product. Reducing fat facilitated a reduction in energy equivalent to 9 kcal per gram.

An important feature of the modified product was a reduction in calories. Sugar (sucrose), traditionally referred to as an essential ingredient in biscuit manufacture, performs several functions, including sweetness, product spread, product colour and eating quality [33]. As sugar levels were decreased, not surprisingly sweetness was reduced because of the decreased sweetness of the sugar substitute used in the modified biscuit. Colour during thermal processing was also reduced and the product became less palatable by loosing its desirable snap or crunch. Like fat reduction, addressing the problems associated with sugar reduction required a complex combination of modifications. Sugar is a bulky raw material and simply removing it, or reducing it from the formulation had the effect of increasing final fat levels. A substitute bulking-ingredient was therefore required, and a mix of polydextrose and fructo-oligosaccharide was found to provide the best results. Of importance here, was the contribution these materials made to further reducing the energy content of the biscuit. Sugar contributes 4 kcal per gram, whilst polydextrose contributes 1 kcal per gram and fructo-oligosaccharide contributes 2 kcal per gram. The addition of both polydextrose and fructo-oligosaccharide made possible the removal of all the sugar in the original biscuit base. In addition, fructo-oligosaccharide is a pre-biotic fibre, and is reported to improve gut health [20,21]. Product sweetness was enhanced by the use of a heat-stable synthetic sweetener (acesulfame K). Product spread and eating quality was improved by increasing the concentration of aerating agent. This had the effect of opening the structure thereby yielding a more palatable product. The required enhancement of surface colour was made possible with the addition of whey powder, which contributes the raw materials required for Maillard Reactions (reducing sugars and amino acids). Maillard Reactions are known to contribute to the surface or crust colour of many baked items. The complete removal of sodium chloride from the formulation presented no problems with respect to dough or product viability. However, sodium is a part of the aerating agent (sodium bicarbonate) hence a small level was present in the final product.

### Modified product functionality

Vitamins B_6_, B_12 _and folic acid were added because of their association with serum homocysteine. Homocysteine is associated with increased risk of CHD [23,24,29,31]. Vitamin C is very heat sensitive, and is known to degrade during thermal processing. It was therefore necessary to use an encapsulated form in order to offer protection during this phase of processing. Difficulty was encountered in establishing levels for addition to the base dough that would deliver the required dosage on consumption of the biscuits. Specifically, folic acid was found to be remarkably heat stable. The very low levels needed for the commercial trials necessitated careful handling on plant, particularly ensuring even distribution during the limited mixing phase. Converting the information gathered in the laboratory to a commercially viable manufacturing experience was not straightforward. Development of the biscuit in the laboratory was completed by hand. In this way, softer dough's than those processed on plant had to be used. This gave "false" information, e.g. the extra water needed to produce the softer dough's was misleading due to increased protein hydration.

In addition, the relative gentle action of hand processing compared to the high forces encountered during rotary moulding, allowed the processing of dough's that could not be processed under commercial conditions. However, laboratory based development did confirm associations between ingredients that proved invaluable. For example, the relationship between dough water and fat levels. Translating the lab-based modifications to the biscuit formulation to commercial manufacture proved difficult initially. Though the dough was dry and crumbly it rotary moulded well giving good extraction. In-weights were quickly achieved, with no tearing of the bases. Final product parameters were in place, including product bulk and moisture levels. The biscuit bases were successfully jammed ready for wrapping. Analysis of the product showed desired levels of all vitamins.

In addition, a reduction in energy from 448 kcal per 100 g to 396 kcal per 100 g was achieved. Even with standard jam (a requirement of the company) the total sugar content was reduced from 32.5% to 22.5%. This sugar content in the biscuit came from the jam only, as the sugar in the base had been completely removed. Total fat was reduced from 16.7% to 7% and sodium was halved, from 0.2% to 0.1%. The formulation used in this study was successful in terms of suitability for commercial process parameters. Problem-free dough extraction through the rotary moulder, is crucial for commercial viability. All product quality requirements were achieved, i.e. surface colour, bulk, spread, and weight. Secondary processing highlighted no problems and the product was wrapped using standard product outputs. The findings from this study are consistent with previously published work on effects of ingredient levels [33-35]. With no sucrose in the base recipe, caramelisation reactions during the baking phase were insufficient to give the desired colour to the biscuit surface. Addition of whey powder has long been used in the food industry to enhance crust colour of a variety of baked products including biscuits, and the improvements to surface colour in this study are consistent with those of others [33].

Dough consistency has also been shown to be crucial in the successful manufacture of rotary moulded biscuits. This study has shown that a significant reduction in dough water is required for operational success under commercial manufacturing conditions. Normally, soft dough's tend to give "tails" (distortion of the dough piece following extraction) when rotary moulded, particularly when they contain high fat levels [36]. In this study however, the dough contains very little fat, so any dough that was considered too soft must have contained high levels of water.

In this study, a healthy biscuit was successfully produced under strict commercial manufacturing conditions, and furthermore the biscuit was acceptable to the consumer. The findings of this study confirmed that traditional high-fat and high-sugar biscuits which are not associated with healthy diets by most consumers can be modified to produce a healthy alternative and can be manufactured under strict commercial conditions. At a time when obesity is a major concern in the UK, and foods high in energy and low in nutrients are linked to the causes of obesity and negative health, the manufacturers of these products are under increasing pressure to produce healthy alternatives. Such developments across a range of products will have a profound affect on the food industry as children and adults alike are becoming increasingly aware of the need to consume a health promoting diet.

## Conclusion

This study has confirmed that traditional high-fat and high-sugar biscuits which are not associated with healthy diets by most consumers can be modified. This modification procedure was performed by the addition of vitamin B_6_, vitamin B_12_, Folic Acid, Vitamin C and Prebiotic fibre, while reducing salt and sugar, thereby converting a traditional food product into a functional one. This resulted in a healthy alternative that is not only acceptable to consumers, but can be manufactured under strict commercial conditions.

## Authors' contributions

Dr Wyndhan Boobier designed the modified biscuit. Dr Julien Steven Baker and Professor Bruce Davies made substantial contribution to study design, conception of data and data analysis and interpretation. Boobier, Baker and Davies gave final manuscript approval.
